# Optimisation of cultivation conditions for *Bacillus velezensis* G7 from mangrove plants and exploration of potential bacteriocins

**DOI:** 10.3389/fphar.2025.1530043

**Published:** 2025-02-26

**Authors:** Yang Li, Haotian Ma, Ruixue Pan, Yuner Long, Yining Zhao, Mengbo Yu, Jinju Peng, Yi Ma

**Affiliations:** College of Coastal Agricultural Sciences, Guangdong Ocean University, Zhanjiang, China

**Keywords:** mangroves, bacteriocin, optimization, *Bacillus velezensis*, culture

## Abstract

**Introduction:**

Bacteriocin P7 was extracted from the cell-free supernatant (CFS) of *Bacillus velezensis* G7, which is a strain isolated from mangrove plants.

**Methods:**

In this study, the culture conditions of *B. velezensis* G7 were optimised using an orthogonal test. The (CFS) was subsequently purified by using TA-GF75 gel chromatography, Tiderose Q HP anion chromatography and reversed-phase high-performance liquid chromatography (RP-HPLC). Finally, the bacteriocin was identified by using LC-MS/MS.

**Results and discussion:**

The optimal culture conditions for *B. velezensis* G7 are 4.5 g/100 mL glucose, 1.5 g/100 mL yeast, and 1.2 g/100 mL MgSO_4_·7H_2_O. The stability of the CFS is affected by several factors, including heat, UV treatment and different storage conditions. High temperatures and long UV irradiation treatments significantly reduce the stability of CFS, which is more sensitive to strong acids, bases and enzymatic degradation. The minimum inhibitory concentration (MIC) of purified bacteriocin P7 against *S. aureus* was determined to be 30.352 μg/mL. On the basis of the results of the haemolytic activity assay, it was concluded that the use of bacteriocin P7 at concentrations equal to or below the 2 × MIC is safe. The addition of organic solvents and inorganic salts did not affect the bacteriocin P7, while the incorporation of SDS could enhance its antimicrobial efficacy. The bacteriocin was subjected to analysis by LC-MS/MS, which revealed that it was similar to the class I bacteriocin amyloliquecidin GF610. The findings of the present study indicate that the endophytic *B. velezensis* G7 from mangrove plant can produce bacteriocins, thereby providing a reference point for the expansion of novel bacteriocin sources.

## 1 Introduction

Mangrove forests are wetland forests composed of saline tree and shrub species distributed between tropical and subtropical land and sea around the world (Friess, 2016). Due to the specialised nature of their habitats, their metabolic pathways different from those of terrestrial microorganisms and are rich in novel metabolites. A fungus, Aspergillus fumigatus JRJ111048, whose metabolites include one new lipid amide 11-methyl-11-hydroxydodecanoic acid amide, was isolated from the leaves of *Acrostichum specioum*, a mangrove plant endemic to Hainan, and this new compound showed strong insecticidal activity against newly hatched larvae of *Bacillus* Species (Islam et al., 2022). Different endophytes are present in mangrove plants and soils, for example, in the mangrove forests of the Andaman Nicobar Islands, India, a total of three *Bacillus* species were found, two of which were identified as *Bacillus subtilis* and *B. velezensis* (Guo et al., 2017). Extracts of mangrove fruits, roots, stems and leaves have been observed to possess antimicrobial, antioxidant, anti-inflammatory and anticancer activities (Acharya et al., 2023). The majority of plants in mangrove ecosystems are angiosperms that produce specific secondary metabolites, which are employed to control and even communicate with phytopathogenic bacteria and fungi. Plants are subjected to attack by phytopathogenic bacteria, fungi and viruses and, in response, produce a substantial number of antimicrobial secondary metabolites (Sulaiman et al., 2022). In one study, a total of 386 strains of *Lactobacillus* spp. were isolated from mangrove forests in southern Thailand. Four strains were selected for screening on the basis of their potential to produce antimicrobial secondary metabolites, specifically bacteriocins, which demonstrated inhibitory activity against *Lactobacillus sakei*, *Listeria monocytogenes* and *Brochothrix thermokilleri* (Hwanhlem et al., 2013). *Bacillus* species may produce bacteriocins that resemble those produced by lactic acid bacteria, such as lantibiotics and pediocin-like bacteriocins. *Bacillus* is widespread in soil, plants, food and the intestines of animals, and its trophozoites divide and multiply approximately every half hour. Changes in environmental factors such as nutrient status, temperature, pH, oxygen content and salt concentration can trigger the transformation between the spores and the nutrients. *Bacillus* is characterised by high temperature resistance, rapid resurrection and strong secretion of enzymes, and can survive under both aerobic and anaerobic conditions. In the lack of nutrients, drought and other conditions to form spores, in the right conditions and can re-emerge into nutrients. *Bacillus* has a large number of strains with special functions, and these strains show a wide range of potential applications in various fields such as livestock and poultry breeding, agriculture and medicine.

Bacteriocins are peptides synthesized by ribosomes with antimicrobial activity (Li et al., 2024). The genus *Bacillus* is the primary source of bacteriocins. Many *Bacillus* species produce a variety of bacteriocins, including *Bacillus thuringiensis*, *Bacillus thermophilus*, *Bacillus licheniformis*, *Bacillus cereus*, *Bacillus amyloliquefaciens*, *B. subtilis*, and *Bacillus coagulans* (Elazzazy et al., 2024). *Bacillus bacterin* has broad-spectrum antimicrobial activity, showing effectiveness against both gram-positive and gram-negative bacterial strains (Bastos et al., 2008; Wang Z. et al., 2024). Bacteriocins from *B. velezensis* significantly inhibited *Streptomyces scabies,* with a minimum inhibitory concentration of 10.58 μg/mL, and were stable to UV radiation and high temperature (Zhao et al., 2022).

The nutrient composition of a medium influences the microbial fermentation process (Gomes et al., 2022). More specifically, small changes in the composition of the fermentation medium can significantly affect the production and metabolic composition of microorganisms (Vlajkov et al., 2022a). To increase the capacity of *Bacillus* to produce bacteriocins, the development of an efficacious medium to augment the production of bacteriocins by *Bacillus* through the screening of carbon, nitrogen, and inorganic salt components is regarded as a viable strategy (Lajis, 2020). Taswar Ahsan (Ahsan et al., 2023) screened *B*. *velezensis* BP-1 fermentation medium by determining 15 g/L crude flour as a carbon source, 13.68 g/L peanut root extract as the nitrogen source, and 0.50 g/L magnesium sulfate as the inorganic salt component of the medium, which led to 90% inhibition of *Peyronellaea arachidicola*. Adequate addition of magnesium sulphate can change the medium from neutral to weakly alkaline, creating a more favourable environment for the growth of certain microorganisms. Mg^2+^ in magnesium sulphate plays an important role in the manufacture of proteins by activating a variety of enzymes and participating in the synthesis of amino acids, the transcription and translation of genes, the production of proteins that are structural components of the ribosome, and other processes (Chen et al., 2025).

**FIGURE 1:**
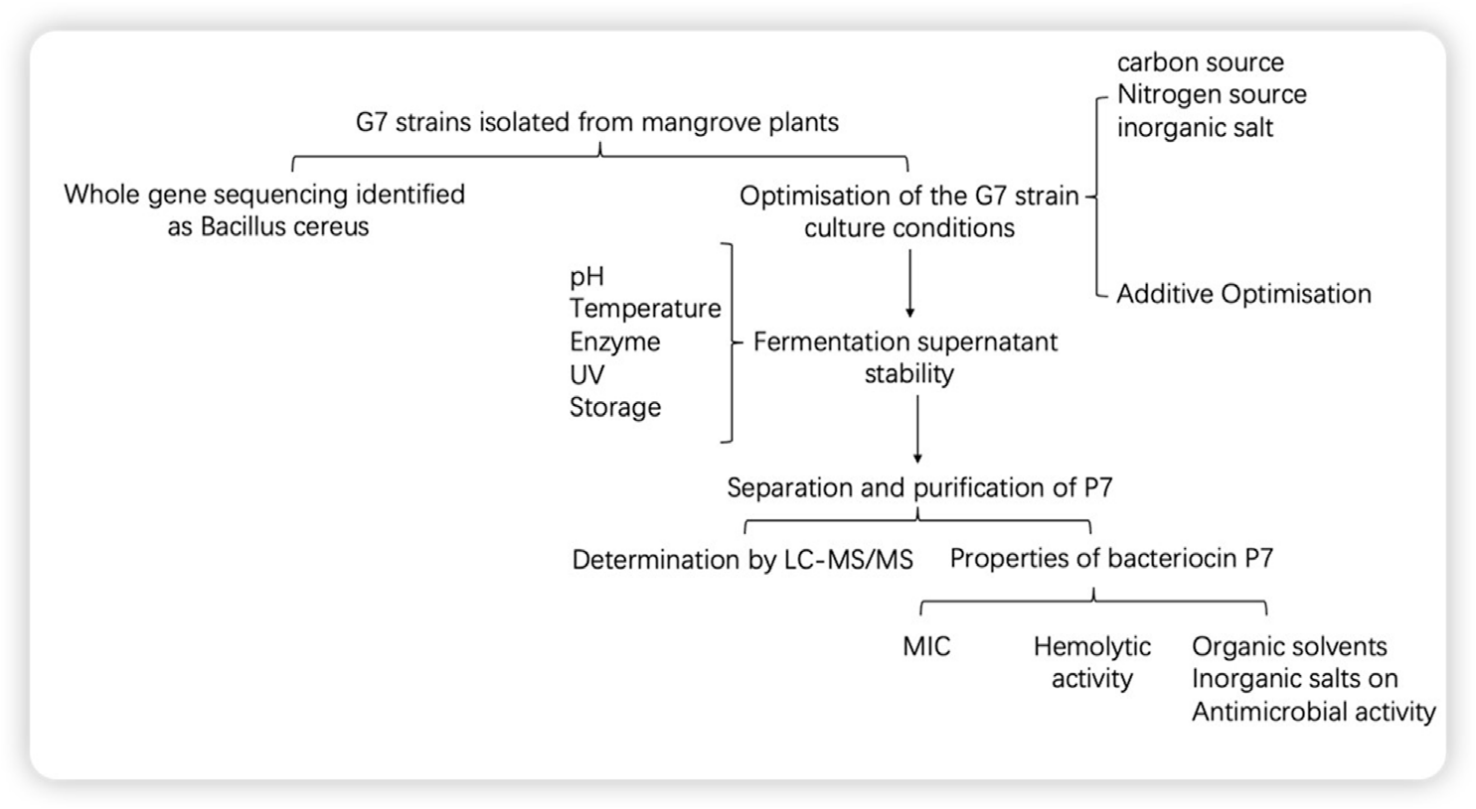
The experimental scheme of this study.

A total of 227 culturable endophytes were isolated from mangrove roots tissues of the species *Avicennia marina* from Zhanjiang Mangrove Nature Reserve, Guangdong Province, China. In this experiment, an endophytic strain G7 was used, which exhibited good antimicrobial activity against both Gram-positive and Gram-negative bacteria, demonstrating broad-spectrum antimicrobial activity, and its culture medium was optimised. The stability and haemolysis of the cell-free supernatant were determined, and the bacteriocin was purified by gel chromatography, ion chromatography and high-performance liquid chromatography. The bacteriocin was also identified by LC-MS/MS. The experimental scheme of this study is shown in Figure 1. These results provide a theoretical basis for the research and application of bacteriocins in food, agriculture, and animal husbandry.

## 2 Materials and methods

### 2.1 Reagents and strains

*B. velezensis* G7 was isolated from Zhanjiang Mangrove Forest Nature Reserve, Guangdong, China. The bacterial strains used in this study, including *Escherichia coli* (CMCC(B)44,103), *Pseudomonas aeruginosa* (ATCC9027), *Staphylococcus aureus* (ATCC25923), and *L. monocytogenes* (ATCC19115), were obtained from Huankai Microbiology Technology Co (Guangzhou, Guangdong, China). All strains were cultured in LB liquid medium and stored at −80 °C with a solution of glycerol at a concentration of 30%. Chicken blood obtained from hongquan bio-tel(Guangzhou, Guangdong, China).

### 2.2 Optimisation of single components of culture media

*B. velezensis* G7 was inoculated in LB liquid medium overnight at 30 °C, then inoculated with 2% of the inoculum into different media and incubated at 180 rpm and 30 °C for 24 h. Following this, the mixture was centrifuged at 8000 rpm and 4 °C for 30 min, after which the supernatant was filtered through a 0.22 μm membrane to obtain the CFS. The extent of bacterial inhibition was determined by measuring the diameter of the inhibition zone formed around the inoculation site on solid agar plates. Specifically, the overnight-cultured indicator bacteria were inoculated into an unconsolidated LB solid medium at an inoculum of 1%, and wells were punched with a sterilised punch. Subsequently, 200 μL of CFS was added to the wells, and the culture was incubated at 37°C for 24 h. Thereafter, the inoculum was filtered through a 0.22 μm filter membrane at 4°C to obtain the CFS.

The carbon source medium consisted of 2 g/100 mL yeast and 1.0 g/100 mL NaCl. Glucose, sucrose, fructose, cyclodextrin, and maltose were added to the basal medium at a concentration of 2 g/100 mL. The nitrogen source medium was supplemented with glucose, the optimal carbon source. The nitrogen source basal medium consisted of 2 g/100 mL glucose and 1.0 g/100 mL NaCl. Yeast, casein, peptone, beef meal, and fish protein at a concentration of 2 g/100 mL were added to the basal medium. The inorganic salt medium consisted of 2 g/100 mL glucose and yeast. Magnesium sulfate heptahydrate, dipotassium hydrogen phosphate, sodium chloride, calcium chloride, and ammonium persulfate were added to the basal medium at a concentration of 1 g/100 mL.

### 2.3 Optimisation of the carbon, nitrogen and inorganic salt contents in culture media

The optimum carbon source, nitrogen source and inorganic salts were selected as the basic medium. To optimize the amount of carbon source additive, concentrations of 1, 2, 3, 4, 5, and 6 g/100 mL glucose were added. To optimize the amount of nitrogen source additive, concentrations of 1, 2, 3, 4, 5, and 6 g/100 mL yeast were added. For the optimization of inorganic salt, MgSO_4_·7H_2_O was added at concentrations of 0.6, 0.8, 1.0, 1.2, 1.4, and 1.6 g/100 mL. The antimicrobial activity of the above culture supernatants was determined under the same experimental conditions, and all three sets of replicates were performed.

### 2.4 Orthogonal test

According to the optimised medium screened by the one-way test, Orthogonal tests were designed by Latin was carried out as shown in Table 1. The three factors were the optimal carbon, nitrogen and inorganic salts screened, the three levels were the optimal additive amounts of the factors, and three-factor and three-level analyses were carried out. The experiment was repeated three times.

**TABLE 1:** Orthogonal experimental design factor level table.

Level	Ingredient		
	Glucose (g/100 mL)	Yeast (g/100 mL)	MgSO_4_·7H_2_O (g/100 mL)
Group 1	1.5	0.5	0.6
Group 2	1.5	1.0	1.2
Group 3	1.5	1.5	1.8
Group 4	3.0	0.5	1.2
Group 5	3.0	1.0	1.8
Group 6	3.0	1.5	0.6
Group 7	4.5	0.5	1.8
Group 8	4.5	1.0	0.6
Group 9	4.5	1.5	1.2
K1	45.31	28.76	45.00
K2	47.19	54.54	49.34
K3	50.33	59.53	48.49
R	5.02	30.77	3.49

### 2.5 Stability of CFS

#### 2.5.1 Acid‒base stability

The treated CFS was divided into five equal parts, adjusted to pH 2, 4, 6, 8 and 10 with 1 mol/L HCl or NaOH, stored in a refrigerator at 4°C for 24 h, and then adjusted back to the natural pH for the detection of antimicrobial activity (Ming et al., 2022).

#### 2.5.2 UV stability

The appropriate amount of treated CFS was divided into five equal portions, the CFS broth was placed under ultraviolet light (30 W) for 10, 20, 40, 60 and 90 min, and then the antimicrobial activity was tested (Ming et al., 2022).

#### 2.5.3 Temperature stability

An appropriate amount of treated CFS was divided into five equal portions, the CFS broth was placed in a water bath at 40, 60, 80, or 100°C for 60 min and 121°C for 20 min, and then the mixture was cooled to room temperature to detect the antimicrobial activity (Ming et al., 2022).

#### 2.5.4 Storage stability

The treated CFS was divided into three equal portions and stored at 4°C, −25°C and −80°C for 0, 3, 6, 9, 12, 18 and 24 days for the detection of antimicrobial activity (Ming et al., 2022).

#### 2.5.5 Protease stability

The appropriate amount of treated CFS was divided into five equal portions, pepsin, trypsin, papain, protease K, and protease E were added to the CFS (1 g/mL), and the antimicrobial activity was detected by heating in a water bath at 37°C for 1 h (Ming et al., 2022).

### 2.6 Isolation and purification of bacteriocin

The optimised culture conditions for *B. velezensis* G7 consisted of a liquid volume of 30 mL, an inoculum volume of 2%, an incubation temperature of 30°C and an incubation time of 24 h. The culture was subsequently centrifuged at 4°C for 30 min at 8000 rpm, and the supernatant was concentrated 10-fold using a rotary evaporator, followed by filtration through a 0.22 μm filter membrane. The concentrated crude extract was loaded onto a deionised water-equilibrated TA-GF75 gel column and subjected to purification at a flow rate of 0.55 mL/min. The obtained fraction with bacteriostatic effects was concentrated and loaded onto a Tiderose Q HP anionic column equilibrated with 1 mol/L NaCl and subjected to anionic column purification at a flow rate of 3 mL/min (Ma et al., 2025). The fraction with bacteriostatic activity was then concentrated and subjected to RP‒HPLC separation and purification. This process consisted of mobile phase A (ddH_2_O+0.1% TFA) and mobile phase A (acetonitrile+0.1% TFA). The elution procedure was as follows: 5% B; 11–20 min, 5%–50% B; 21–30 min, 50%–70% B; and 31–38 min, 70%–95% B. The bacteriostatic activity of the isolated bacteriocin was detected via the solid agar perforation method and the micro broth twofold dilution method. The bacteriocins were concentrated and stored at −80°C for later use. The molecular weights of the bacteriocins were determined via Tricine-SDS‒PAGE. The gels were electrophoresed at 30 V for 1 h and then electrophoresed at 100 V, after which the gels were stained with Coomassie blue (Peng et al., 2024).

### 2.7 LC‒MS/MS

Qingdao Stantec Standard Testing Company, Shandong, China, conducted the LC‒MS/MS analysis of bacteriocin P7 (Selvam et al., 2021). The sample was processed before mass spectrometry analysis: total bacteriocin was extracted, enzymatically digested, and desalted, and the supernatant was left. Separation was carried out on a chromatographic column with an injection volume of 1 μL. Equilibration was performed with 92% liquid A (0.1% formic acid aqueous solution). The relevant liquid‒phase gradient was set as follows: 0–98 min, the linear gradient of liquid B (0.1% formic acid acetonitrile aqueous solution (80% acetonitrile)) was from 8% to 28%; 98–113 min, the linear gradient of liquid B was from 28% to 37%; 113–117 min, the linear gradient of liquid B was from 37% to 100%; and 117–---- 120 min, liquid B was maintained at 100%. Mass spectrometry analysis was performed on a Thermo QE HF mass spectrometer (Thermo Fisher) with an analysis duration of 120 min. Detection mode: positive ions. The mass‒charge ratios of the peptides and fragments of the peptides were determined according to the following method: 20 fragment profiles were collected after each full scan (MS2 scan). The scanning range was 400–1800, the primary resolution was 60,000, the secondary resolution was 15,000, and the collision energy was CE28eV. The bacteriocin identification results were obtained by searching the corresponding databases with Proteome Discoverer 2.5 (Banerjee et al., 2017).

### 2.8 Analysis of the bacteriocin propertie

#### 2.8.1 Minimum inhibitory concentration (MIC)

The MIC was determined via a twofold micro broth dilution method. One hundred microlitres of *S. aureus* were diluted 1 × 10^−3^ times with LB, 100 μL of diluted *S. aureus* was added to 96-well plates, and then 100 μL of bacteriocin was serially diluted in 96-well plates containing indicator bacteria. At the same time, the wells containing bacteriocin and indicator bacteria were used as the control, and bacterial growth was observed after 16 h at 37°C. As a control, bacterial growth was observed at 37°C for 16 h. The lowest concentration at which the medium was clear to the naked eye and no bacterial growth was observed was judged to be the lowest inhibitory concentration of the drug (Banerjee et al., 2017).

#### 2.8.2 Haemolytic effects of bacteriocin P7

The appropriate amount of domestic chicken blood was taken and centrifuged at 4 °C and 1000 × g for 10 min to obtain erythrocytes, which were washed three times with PBS, and the erythrocytes were fully suspended during the washing process. At the same time, the fragmentation of erythrocytes was avoided as much as possible, and the erythrocytes were subsequently resuspended in PBS. Different concentrations of bacteriocin solution (1 × MIC, 2 × MIC, and 4 × MIC) were mixed with an equal volume of erythrocyte suspension, added to a 2 mL centrifuge tube and incubated at 37°C for 1 h. The mixture was centrifuged at 4°C and 1000 × g for 10 min to obtain the supernatant, 100 μL was added to a 96-well plate, and an enzyme marker was used to measure the absorbance at 540 nm. Under the same treatment conditions, 0.1% Triton X-100 and PBS were used as positive controls and blank controls, respectively. The OD_540_ value of the PBS-treated erythrocyte solution was considered 0% haemolysis, and the OD_540_ value of the 0.1% Triton X-100-treated erythrocyte solution was considered 100% haemolysis (Selvam et al., 2021; Banerjee et al., 2017). The formula for calculating the haemolysis rate of bacteriocins is as follows:

Haemolysisrate(%)=100×[(A−A1)/(At−A1)]

where A is the OD_540_ value of blood cells after treatment with bacteriocin, A1 is the OD540 value of blood cells after treatment with PBS, and At is the OD_540_ value of blood cells after treatment with Triton X-100.

#### 2.8.3 Effects of different chemicals on the bacteriostatic activity of bacteriocin P7

The bacteriocin P7 was divided into five equal portions (1 mL) and mixed with EDTA, SDS, CO(NH_2_)_2_ and 25% CH_3_OH and 25% C_2_H_5_OH at a mass ratio of 1%. After 2 h at room temperature (25–27°C), the bacteriostatic activity of the mixture was determined via the solid agar perforation method with *S. aureus* used as an indicator organism, and the bacteriocin mixture not treated with the chemical agents was used as a blank control (Banerjee et al., 2017).

#### 2.8.4 Effects of different inorganic salts on the bacteriostatic activity of bacteriocin P7

The bacteriocin was divided into five equal portions (1 mL), and 10 μL of NaCl, CaCl_2_, KCl, ZnSO_4_, or MgCl_2_ at a concentration of 2 mol/L was added and mixed. Bacteriocin without inorganic salts was used as a control, and the inhibitory activity of the mixtures was measured by solid agar perforation to test the effect of the inhibitory activity of the bacteriocin via different metal ions.

## 3 Results

### 3.1 1Identification of strain G7

**FIGURE 2:**
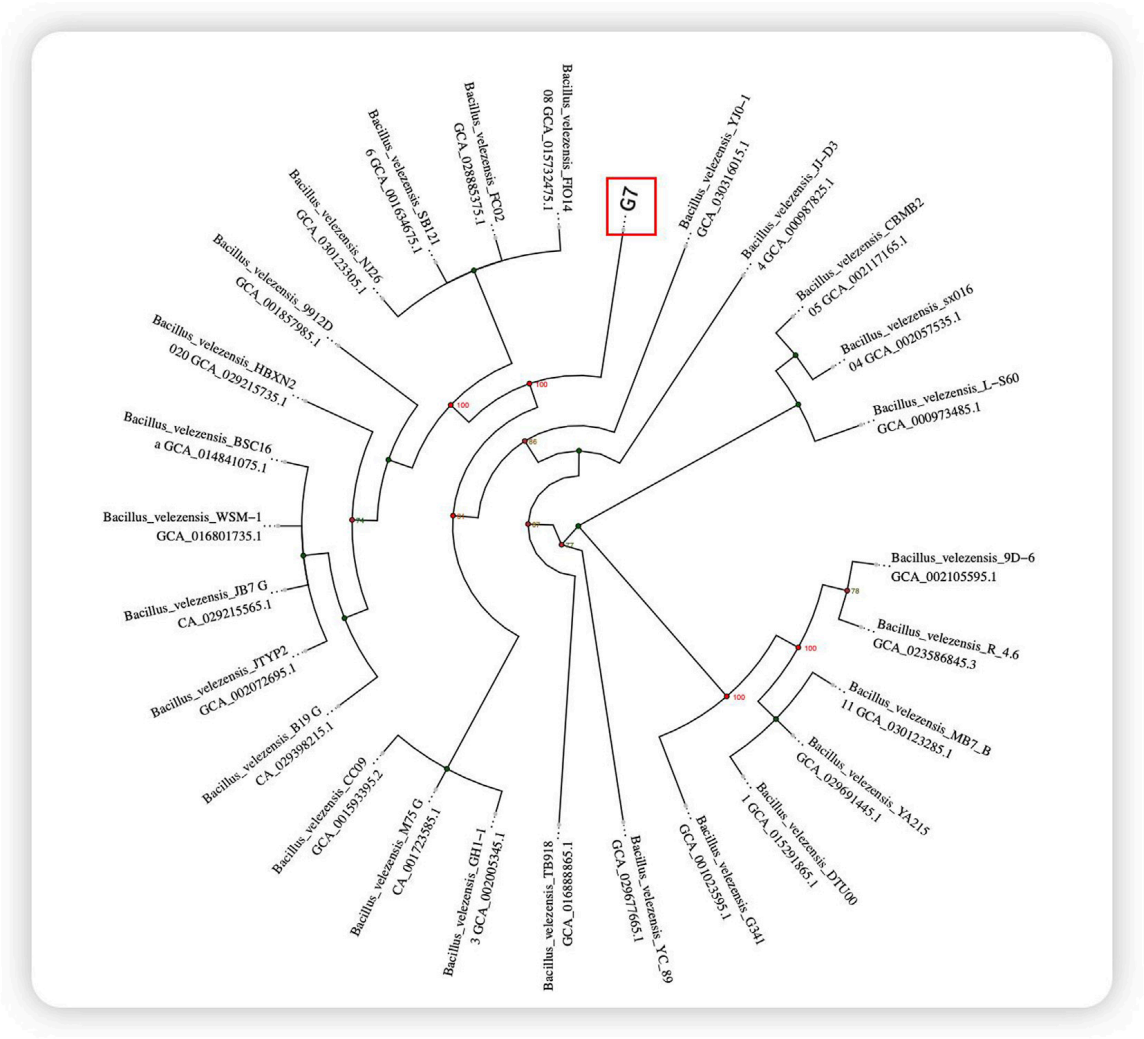
Circular evolutionary tree based on 16S rRNA.

A strain with remarkable bacterial inhibition was screened, and a total of 857 bacterial strains were isolated from the mangrove roots. Among these strains, G7 exhibited exceptional bacteriostatic activity. The 16S rRNA gene sequence of G7 was amplified through PCR and compared with several *B*. *velezensis* strains, revealing a high degree of similarity (Figure 2). Consequently, the isolate was designated *B. velezensis* G7.

### 3.2 Screening of single components of culture media

**FIGURE 3:**
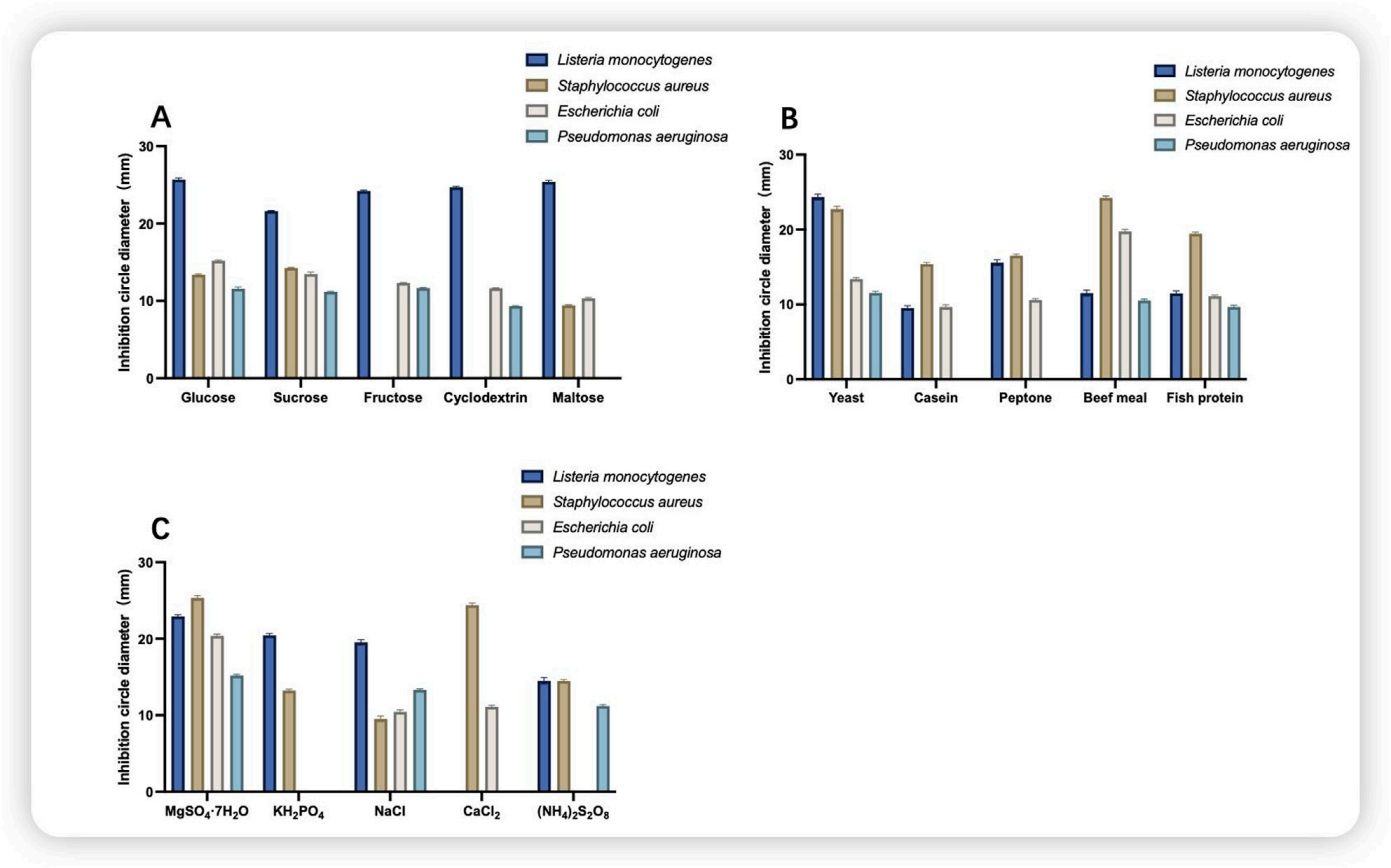
Inhibitory effects of different components of the medium. **(A)** Comparison of the size of the circle of inhibition on different carbon sources; **(B)** comparison of the size of the circle of inhibition on different nitrogen sources; **(C)** comparison of the size of the circle of inhibition on different inorganic salts.

Figure 3 shows the single-component screening of the medium, which comprises glucose as a single carbon source, yeast as a single nitrogen source, and MgSO_4_·7H_2_O as a single inorganic salt. The antimicrobial agents were effective against all four indicator bacteria, with the greatest inhibition observed against *S. aureus* and *L. monocytogenes*, both of which were greater than 20 mm in diameter.

### 3.3 Optimisation of the amount of each ingredient added

**FIGURE 4:**
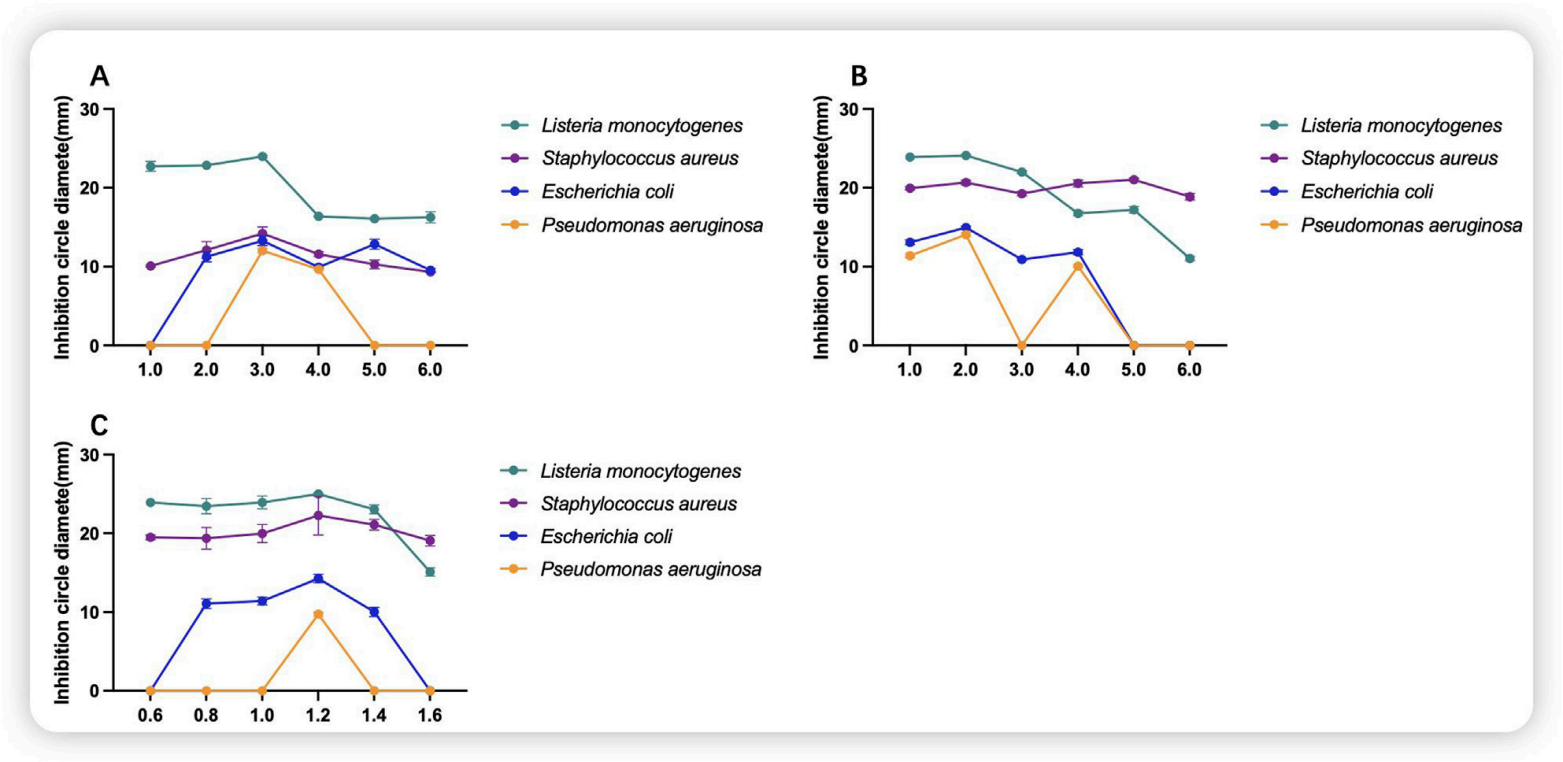
Inhibitory effects of component additions to the culture medium. **(A)** Glucose; **(B)** yeast; **(C)** MgSO_4_·7H_2_O; (g/100 mL).

As shown in Figure 4A, glucose at the right concentration will have a certain inhibitory effect on the indicator bacteria, and the inhibitory effect may be deteriorated by using too much, and the inhibitory effect on the indicator bacteria will be most obvious when the concentration of glucose is 3.0 g/100 mL. As shown in Figure 4B, the inhibitory effect on the growth of indicator bacteria was more pronounced at a yeast concentration of 2.0 g/100 mL, and disappeared as the concentration was increased to 6.0 g/100 mL for the growth of *P. aeruginosa and E. coli*. As shown in Figure 4C, when the MgSO_4_·7H_2_O concentration was 1.2 g/100 mL, the most pronounced inhibitory effect was observed. Consequently, this concentration was selected as the inorganic salt concentration for the medium.

### 3.4 Orthogonal test results

The results of the one-factor level test indicated that glucose, yeast and MgSO_4_·7H_2_O were the components of the fermentation medium. Latin software was employed to select three factors and three levels for orthogonal test optimisation, and the resulting level scheme is presented in Table 1. As shown in Tables 1–3, as a component of the medium, yeast was the main influencing factor followed by glucose and magnesium sulphate heptahydrate. According to the comparison between K values, the most pronounced inhibitory effect was observed in group 9. Therefore, the optimal culture conditions for strain G7 were identified as 4.5 g/100 mL glucose, 1.5 g/100 mL yeast, and 1.2 g/100 mL MgSO_4_·7H_2_O.

**TABLE 2:** Inhibitory effects of different fermentation broths in the orthogonal test.

	Indicator bacteria				
		*Staphylococcus aureus*	*Listeria monocytogenes*	*Escherichia coli*	*Pseudomonas aeruginosa*
Inhibition circle diameter (mm)	Group 1	13.97 ± 0.12	16.11 ± 0.2	—	—
	Group 2	20.82 ± 0.57	18.34 ± 0.52	10.4 ± 0.82	—
	Group 3	22.65 ± 0.31	21.35 ± 0.40	12.28 ± 0.36	—
	Group 4	—	17.3 ± 0.30	—	10.95 ± 0.45
	Group 5	20.41 ± 0.40	19.08 ± 0.45	11.98 ± 0.39	9.75 ± 0.25
	Group 6	19.59 ± 0.45	20.05 ± 0.49	12.45 ± 0.77	—
	Group 7	—	16.98 ± 0.27	—	10.98 ± 0.54
	Group 8	22.90 ± 0.13	17.80 ± 0.40	12.13 ± 0.20	—
	Group 9	23.90 ± 0.21	22.65 ± 0.29	13.76 ± 0.21	9.90 ± 0.19

**TABLE 3:** Analysis of variance table (ANOVA).

Factor	DV	TSS	df	MS	F	P-value
Glucose	*Staphylococcus aureus*	51.511	2	25.756	0.913	0.523
	*Listeria monocytogenes*	0.450	2	0.225	0.441	0.694
	*Escherichia coli*	1.722	2	0.861	1.234	0.448
	*Pseudomonas aeruginosa*	94.671	2	47.336	5.454	0.155
Yeast	*Staphylococcus aureus*	582.419	2	291.210	10.322	0.088
	*Listeria monocytogenes*	31.988	2	15.994	31.295	0.31
	*Escherichia coli*	298.696	2	149.348	214.126	0.005
	*Pseudomonas aeruginosa*	30.972	2	15.486	1.784	0.359
MgSO_4_·7H_2_O	*Staphylococcus aureus*	35.571	2	17.786	0.630	0.613
	*Listeria monocytogenes*	3.492	2	1.746	3.416	0.226
	*Escherichia coli*	0.032	2	0.016	0.023	0.978
	*Pseudomonas aeruginosa*	94.698	2	47.349	5.456	0.155
SEM	*Staphylococcus aureus*	3037.612	2	28.212		
	*Listeria monocytogenes*	3235.232	2	0.511		
	*Escherichia coli*	893.956	2	0.697		
	*Pseudomonas aeruginosa*	427.037	2	8.679		

### 3.5 Stability results of fermentation broth

**FIGURE 5:**
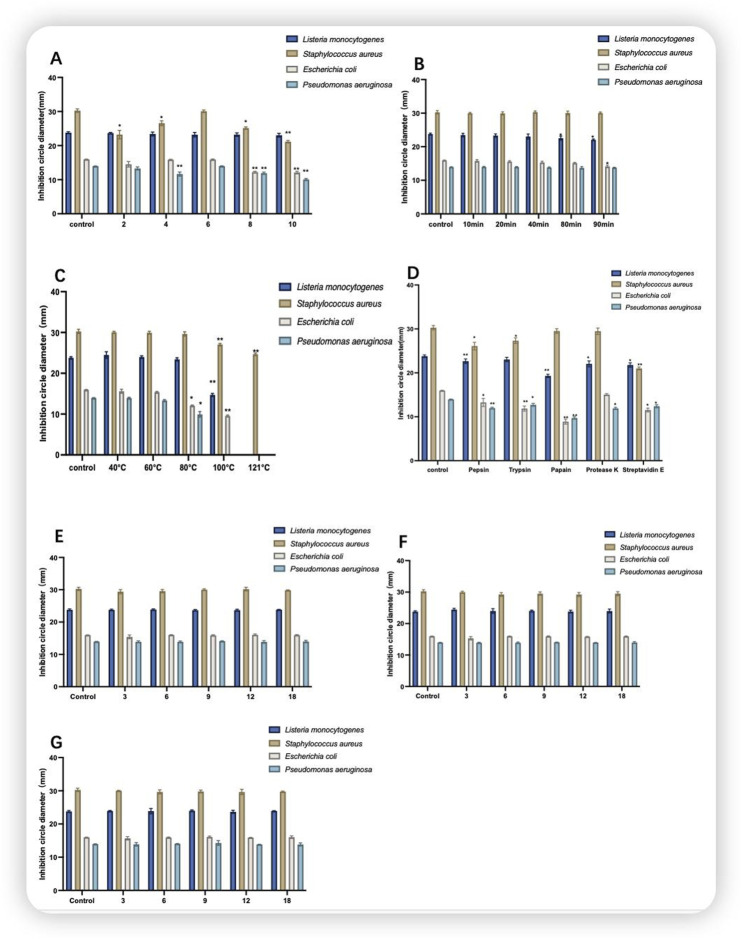
Stability of the cell-free supernatant. **(A)** Acid-base stability; **(B)** UV stability; **(C)** temperature stability; **(D)** enzyme stability; **(E)** 4°C storage stability; **(F)** −20°C storage stability; **(G)** −80°C storage stability; where Control is the control condition; note that *indicates a significant difference (P < 0.05) and **indicates a highly significant difference (P < 0.01).

The pH of the control protein was 6.5. As shown in Figure 5A, different pH values affected the antimicrobial activity of the CFS for all four indicator bacteria. Among pH 8 and 9, the size of the circle of inhibition of CFS against *E. coli* (control: 15.96 ± 0.66) was 12.24 ± 0.24 and 12.12 ± 0.38, and the size of the circle of inhibition of CFS against *P. aeruginosa* (control: 13.96 ± 0.06) was 11.92 ± 0.33 and 10.05 ± 0.28. Compared with that of the control, and the antimicrobial activity was highly significantly reduced (P < 0.01). As shown in Figure 5B, compared withthe control group, the antimicrobial activity of CFS against *L*.*monocytogenes* (control:23.78 ± 0.29) decreased significantly (P < 0.05) with increasing UV irradiation time with a circle of inhibition size of 22.56 ± 0.59 after 80 min and against *E. coli* after 90 min (P < 0.05) with a circle of inhibition size of 14.14 ± 0.39. CFS was heated in a water bath at 40–100°C for 60 min and autoclave at 121°C for 20 min before observing the change in antimicrobial activity. As shown in Figure 5C, the CFS was stable at 40–60 °C, with no significant difference. With increasing temperature, the antimicrobial activity of the CFS against *E*. *coli* and *P. aeruginosa* decreased significantly at 80 °C (P < 0.05), and at 100 °C, there was no antimicrobial activity against *P. aeruginosa*. At 120 °C, only antimicrobial activity against *S. aureus* was detected. As shown in Figure 5D, the control group was not subjected to enzyme treatment. compared with that of the control group, the antimicrobial activity of the culture broth treated with trypsin against *E*. *coli* decreased. The antimicrobial activity of the culture broth treated with papain showed a decline against *L. monocytogenes*, *E*. *coli*, and *P. aeruginosa*. The antimicrobial activity of the culture broth treated with pepsin and protease E against *E*. *coli*, *P. aeruginosa*, *S. aureus* and *L. monocytogenes* decreased. As shown in Figures 5E–G, the antimicrobial activity of the CFS stored at 4°C, −20°C and −80°C for 0–18 days remained relatively stable compared with that of the control group.

### 3.6 Purification of bacteriocin P7

**FIGURE 6:**
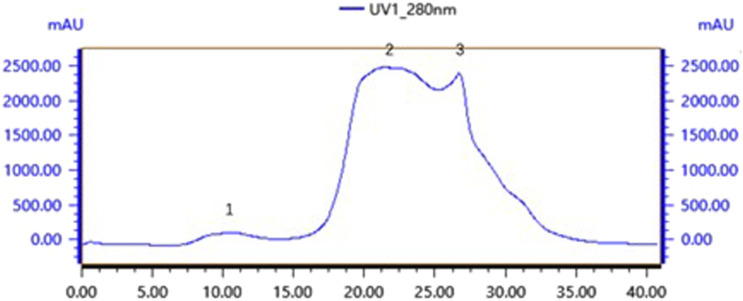
TA-GF75 gel chromatography.

**FIGURE 7:**
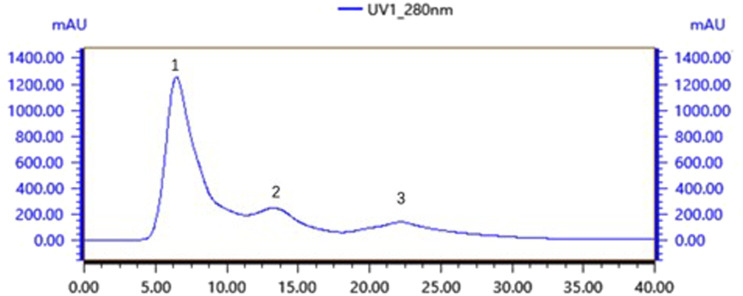
Tiderose Q HP anion chromatography.

**FIGURE 8:**
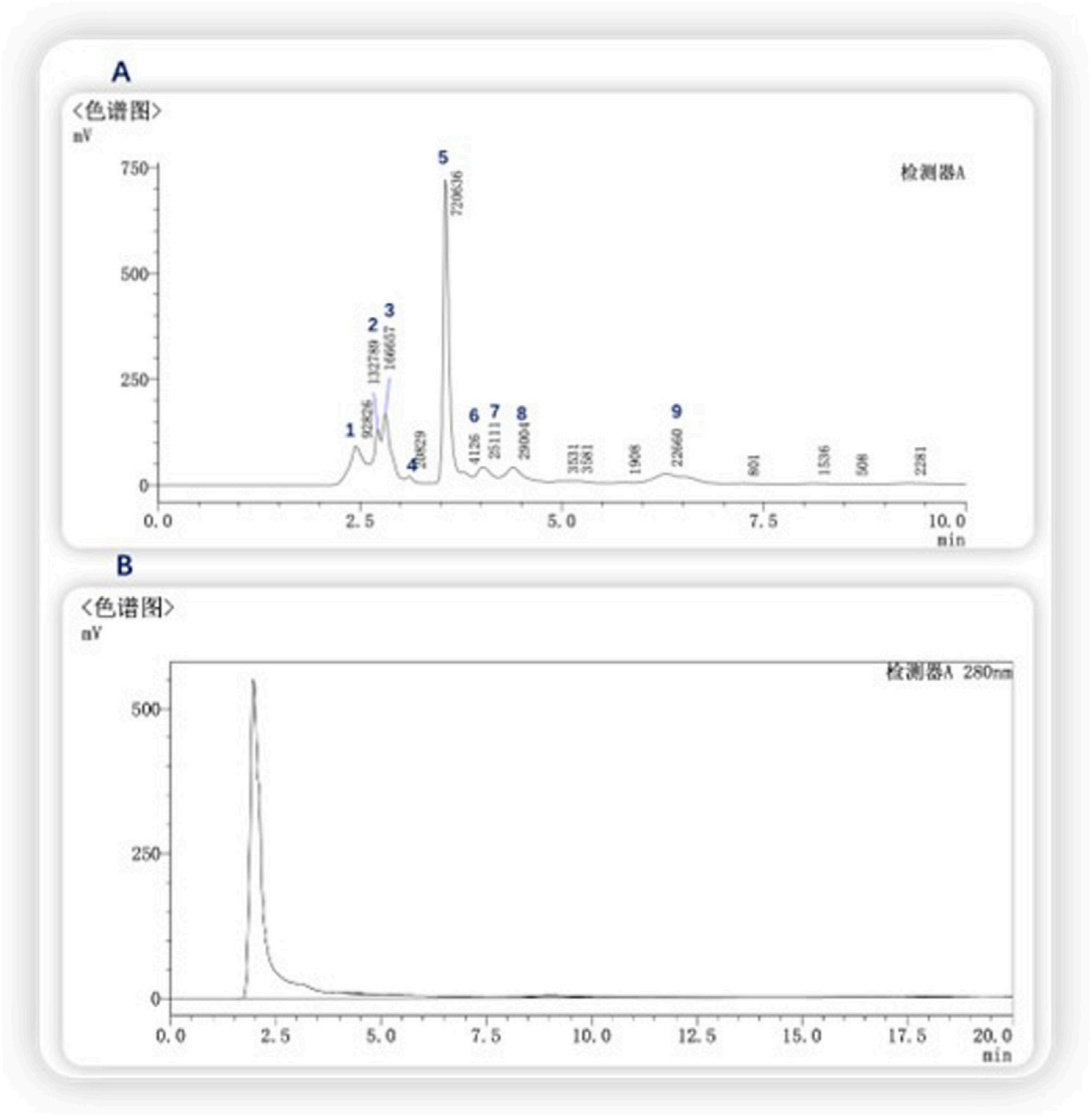
RP-HPLC purification **(A)** First isolation and purification **(B)** Second isolation and purification.

Gel chromatography separates molecules based on their size; within the column, molecules larger than the gel voids are excluded and left directly out of the column, small molecules seep into the gel voids, and medium-sized molecules fall somewhere in between. The main components of the gel column packing GF75 used in this experiment are highly cross-linked agarose and dextran. The separation range for linear molecules is 0.5kd∼30kd, for spherical molecules 3kd∼70kd, and for nucleic acids less than 50bp. Following gel chromatography of the CFSs via the TA-GF75 method, three distinct peaks were obtained. As shown in Figure 6, the second peak exhibited notable bacteriostatic efficacy. The fraction corresponding to the second peak was concentrated and subjected to further purification via Tiderose Q HP anion chromatography to finalise the three peaks. Ion chromatography controls the adsorption and desorption of various ionic substances on the surface of the resin to achieve the separation and detection of ions. As shown in Figure 7, the initial peak exhibited notable bacteriostatic efficacy. The fraction collected and corresponding to the initial peak was subsequently concentrated and purified via RP-HPLC, the working principle is that each component of the mixture has a different magnitude and strength of interaction between the mobile and stationary phases, resulting in different retention times in the stationary phase, which sequentially flow out of the column and into the detector for detection. As shown in Figure 8A, Three different peaks appeared, with the initial peak showing significant antimicrobial activity. We collected the initial peak and then purified it again by RP-HPLC as shown in Figure 8B as a single peak.

**FIGURE 9:**
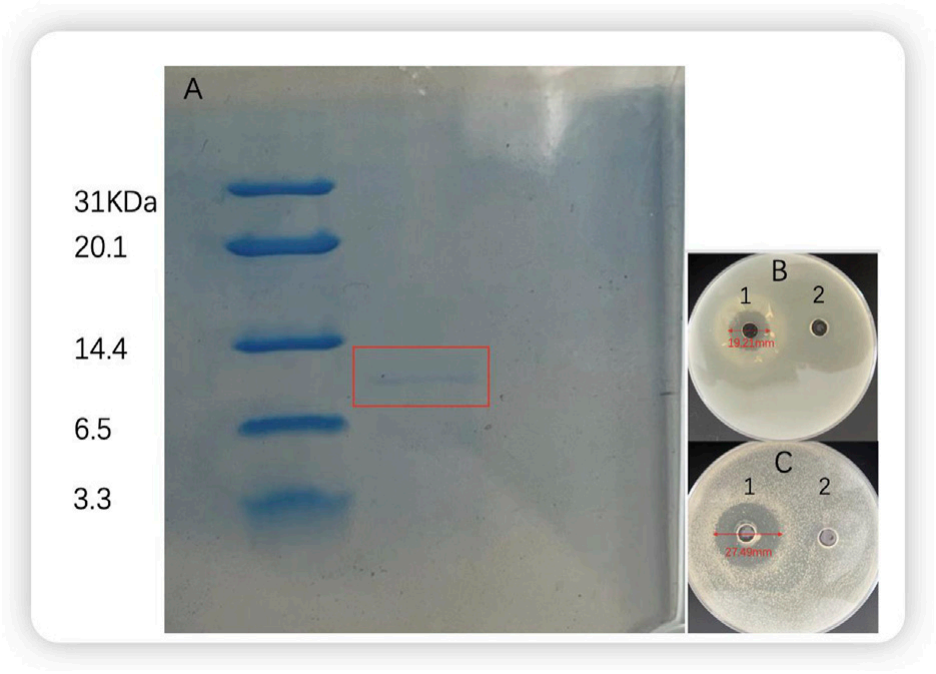
Gel electrophoresis and antibacterial activity of bacterial P7 **(A)** Bacterial P7 gel electrophoresis. M: marker; 1: bacteriocin P7; **(B)** *Listeria monocytogenes* **(C)** *Staphylococcus aureus* 1: bacteriocin P7; 2: blank control.

Furthermore, tricine-SDS‒PAGE analysis of bacteriocin P7 revealed that its molecular weight fell within the range of 6.5–14.4 KDa. The antimicrobial activity of the protein was measured by solid agar perforation method, The same research method was used by Haotian Ma (Ma et al., 2025) and Jinju Peng (Peng et al., 2024). The diameters of the antimicrobial circles of the protein (60.704 μg/mL) against *S. aureus* and *L. monocytogenes* were 27.49 and 19.21 mm, respectively (Figure 9).

### 3.7 LC‒MS/MS

The results were subjected to analysis via LC‒MS/MS and are presented in Table 4. On the basis of the coverage and relative molecular weight of the bacteriocin, it was postulated that the substance should be a class I bacteriocin, similar to amyloliquecidin GF610. This bacteriocin was shown to be active against *L. monocytogenes*, *Clostridium perfringens*, *Clostridium difficile*, *S. aureus* and *Bacillus acidophilus*, with MICs ranging from 0.5 to 7.0 μmol/L,the mic against *S. aureus* was 10 umol/L (Gerst et al., 2022). Bacteriocin p7 isolated in the present study had similar antimicrobial activity and was more effective against *S. aureus* and *L. monocytogenes*, where the mic against *S. aureus* was 30.352 μg/mL. Consequently, this bacteriocin is considered a novel bacteriocin with similarities to amyloliquecidin GF610.

**TABLE 4:** Identification of several proteins by LC-MS/MS.

Protein name	Sequence	Coverage [%]	MW [kDa]
Single-stranded	MKPASKKRGGYGLFNHVMLVGRLTKDPELRFTSAGIPVAHITLAVNRNFKSA		
DNA-binding protein	SGETGTDFVNCTIWRKNAENTALYCQKGSMVGVSGRIQTRSYEKTDGVKVYVTEVMADTVRFMDQKRKEPLAE	5	13.9
YfzA-like protein	MDNEMDKVNKKRPLYKKGWFLTVLAFLVSQLYFNFAELTGWGPNYREMNG	7	12.1
	FPANIAELDFFQTYLSFYDNPWFNIVTVFLGVFTVIQIITGITKDIRNESNNF		
Small ribosomal subunit protein	MKHMPNIKSAIKRTKTNNERRAHNATIKSAMRTAIKQVEASVANNEADKAKT	9	9.9
	ALSEAAKRIDKAVKTGLVHKNAAARYKSRLAKQVNGLSA		
Amyloliquecidin GF610	MKGGDIMSNREKAELYRNASKRTELGFVNPVGEVSEDELRNLAGAADVTPHT	13	8.1
	TPSSLPCGVFVTAAFCPSTKCTSSC		
Fur-regulated basic protein	MAPLLREAINRKKQHLRTKLIRSGFYQDHVQELSGYTLSELEKEYEAVKRLKKAELH	11	6.8
Small, acid-soluble spore protein	MSFFNKEKGKNSDKNKNVIQGALEDAGAALKDDPLQEAVQKKKNNR	17	5.1
DUF3941 domain-containing protein	MKDDDKKPLDNNAVNQKKNRLAEKNRQAGKNQNSKKPDHL	15	4.6

### 3.8 Physicochemical properties of bacteriocin P7

**FIGURE 10:**
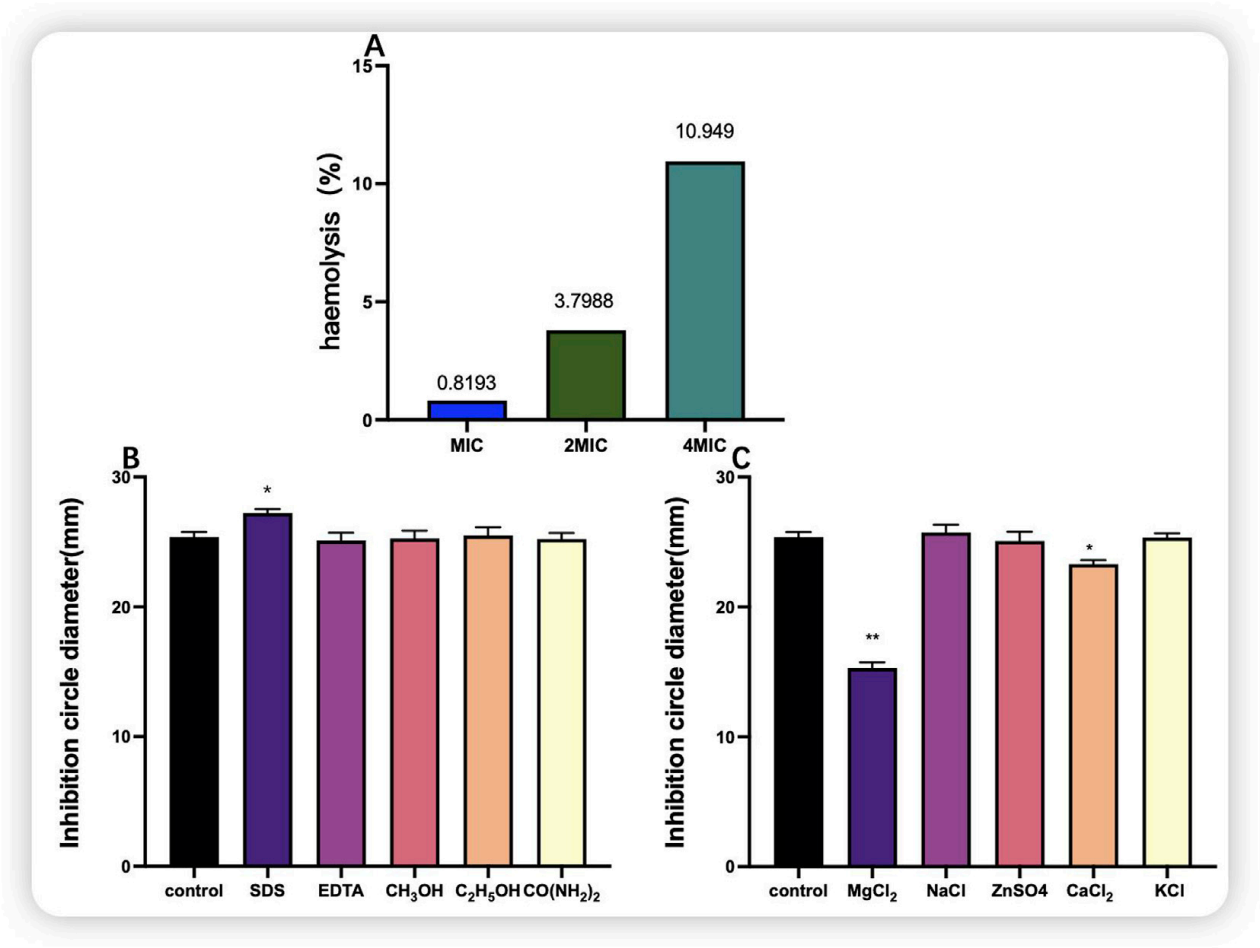
Haemolytic activity of bacteriocin P7 and the effects of organic solvents and inorganic salts on bacteriocin P7. **(A)** Haemolytic activity; **(B)** organic solvent; **(C)** inorganic salt. Note: *indicates a significant difference (P < 0.05), **indicates a highly significant difference (P < 0.01).

The MIC of purified bacteriocin P7 against *S. aureus* was determined to be 30.352 μg/mL. As shown in Figure 10A, the haemolytic activity of the bacteriocin at concentrations of MIC-2 × MIC was less than 10%, and at a concentration of 4 × MIC, the haemolytic activity was 10.949%, which was slightly toxic. Overall, the concentrations of the MIC-2 × MIC bacteriocin are safe. As illustrated in Figure 10B, the five organic solvents had no notable effect on the antimicrobial activity. Interestingly, organic solvents such as SDS increased the inhibitory activity of bacteriocin P7. As shown in Figure 10C, compared with the control group, the MgCl_2_ and CaCl_2_ groups presented highly significant (P < 0.05) and significant (P < 0.01) reductions in the antimicrobial activity of bacteriocin P7, respectively. In contrast, the remaining substances had no significant effect.

## 4 Discussion

Bacteriocins are small peptides synthesized by ribosomes. They are secreted to inhibit the growth of competing bacteria, fungi and some parasites. Therefore, bacteriocins have potential applications in food preservation, animal husbandry, agriculture, and medicine (Fernandes and Jobby, 2022). In the food industry, bacteriocins are always used as natural preservatives to extend the shelf life of food and to prevent spoilage of dairy products. Nisin is the first FDA-approved bacteriocin that has been used in the preservation of pasteurized processed cheese spread (Lahiri et al., 2022). Also, some bacteriocins are used in animal clinical studies and experiments, for instance, the therapeutic efficacy of pyocins had been confirmed in a murine model of *P*. *aeruginosa* sepsis (Six et al., 2021).The composition of the medium has a very large effect on bacteriocin production and bacterial growth, so the production medium needs to be optimised to maximise metabolite production. Carbon is the most important medium component, and the rate of metabolism of carbon sources usually affects the formation of biomass and the production of primary or secondary metabolites (Wang et al., 2018). In this study, glucose was chosen as the carbon source of the fermentation medium, with better antimicrobial activity, and the best effect was achieved at a concentration of 4.5 g/mL, and its antimicrobial effect was affected by either too high or too low concentration. Similar outcomes were achieved by Chang Qing Zhao (Zhao et al., 2017), who optimised the medium for the production of microbial flocculants from *B. subtilis* and identified 20 g/L glucose as the most effective carbon source. The selection of carbon sources as key macronutrients for the growth and reproduction of productive microorganisms and their metabolic activities is considered the first step in the optimisation of culture media. The assimilative capacity of certain carbon sources, as well as their nature and origin, affects biomass growth as well as the type and yield of metabolites (Vlajkov et al., 2022b).

In terms of nitrogen sources, yeast resulted in higher production of bacteriocin P7. This may be because microorganisms can utilise inorganic or organic nitrogen sources. In some cases, the use of specific amino acids can increase productivity; in contrast, unsuitable amino acids may inhibit the synthesis of secondary metabolites (Singh et al., 2017). In the present study, when casein was used as a nitrogen source, the antimicrobial activity of strain G7 was decreased and activity against *P. aeruginosa* disappeared.

Moreover, MgSO_4_·7H_2_O was chosen as the inorganic salt component of this medium and was able to increase the expression of bacteriocin P7. Mg^2+^ is involved in a variety of physiological roles, such as signalling, energy metabolism, fatty acid synthesis, ribosome stabilisation and protein synthesis. The effects of many of these metal ions on the physiological activity of microorganisms are concentration dependent, with low concentrations often showing stimulatory effects and high concentrations showing inhibitory effects (De Leersnyder et al., 2018). Fakher Kamoun (Kamoun et al., 2009) optimized the culture conditions with a carbon/nitrogen ratio of 9, which resulted in more than a fourfold increase in bacteriocin production compared with the use of TSB medium. Yonghong Li (Li et al., 2022) optimised the formulation of the medium by flask fermentation, and *Clostridium butyricum DL-1* reached a viable count of 1.5 × 10^8^ cfu/mL, which was 375 times greater than that of the initial medium culture. The spore production rate was 92.6%. Therefore, the maximum product concentration can be achieved by media optimisation.

*Bacillus* species are capable of producing endospores that exhibit exceptional resilience to unfavourable conditions. These endospores display high-temperature resistance, rapid resurrection, and the secretion of enzymes, enabling the species to survive in both aerobic and anaerobic environments (Owusu-Darko et al., 2020). As a consequence, the CFS of *B. velezensis* G7 in this study was stable at temperatures between 40°C and 60°C. However, a notable decline in antimicrobial activity was observed following an increase in temperature to 80°C. The antimicrobial activity of CFS treated at 80°C was reduced compared to the control, but it was able to maintain its activity for 60min at this temperature, and the antimicrobial activity of CFS at 80°C was reduced, which indicates that its antimicrobial activity could be preserved after a batch pasteurization process (63°C–30 min) or an HTST (High Temperature Short Time) continuous flow pasteurization (72°C–15 s) in dairy products. Moreover, liquid egg products undergo heat treatments at specific times (3.55–6.2 min) and temperatures (55.6–63.3°C). Therefore, these treatments would not affect the antibacterial activity of CFS since temperatures applied are below 80 °C (León Madrazo et al., 2022).

Furthermore, the antimicrobial activity of the CFS was diminished in the presence of UV treatment under various conditions. This may be attributed to the potential inactivation of certain antimicrobial substances resulting from elevated temperatures and UV exposure (Ming et al., 2022). UV has bactericidal and sporicidal properties. The main target of UV damage in microbial cells is DNA, and UV light can act directly on DNA molecules in cells, causing neighbouring pyrimidines on the same DNA strand to form thymine dimers, which leads to changes in the structure of double-stranded DNA, and affects the normal replication of DNA, and ultimately leads to cell death (Begyn et al., 2020). Melanin has been reported to act as a UV-protectant, possibly by absorbing radiation before penetrating the DNA in the spore core or protoplast. melanin synthesis can also be induced by mutations, metal ions and amino acids (Idris et al., 2024). Organic substances and compounds are essential to reduce the negative effects of UV radiation on *Bacillus* cells. TiO2 nanoparticle mixture (anatase and rutile) increase the persistence of *B. thuringiensis* to the UV radiation (Jalali et al., 2020).

CFS exhibited the highest activity at pH 6 and demonstrated a decrease in inhibitory activity in acidic and alkaline environments. However, it maintained more than 50% activity between pH 2 and 10, which may be attributed to the diverse antimicrobial substances produced by different *Bacillus* species. For example, *Lactobacillus rhamnosus* LS8, which was studied by Lihui Zhang et al. (Wang et al., 2022), was only active under acidic conditions. CFS was found to be sensitive to pepsin, protease E, proteinase K and trypsin, as determined by an enzyme stability assay, indicating that the antimicrobial substances produced by *B. velezensis* G7 are natural proteins. The antimicrobial substance produced by *B. velezensis* is sensitive to pepsin, papain, trypsin, proteinase k, and protease E. The antimicrobial substance has also been confirmed to be a peptide (Wayah and Philip, 2018). The CFS of *Pediococcus pentosaceus* LB44 is also sensitive to proteinase K, papain and trypsin (Kaur and Tiwari, 2018). *Bacillus* secretes one or more bacteriostatic active substances during metabolism, and bacteriocin, as one of its secondary metabolites, has the advantages of a wide range of bacteriostatic inhibition, stable physicochemical properties, and excellent safety (Wang H. et al., 2024). *B. velezensis* bacteriocins have a wider range of bactericidal activity than other bacteriocins and are active against both gram-positive and gram-negative bacteria (Vaca et al., 2022).

The haemolytic activity of bacteriocin P7 produced by *B. velezensis* G7 was determined, and it was found that the haemolysis rate was less than 5% at concentrations in the MIC-2MIC range. Therefore, it can be concluded that bacteriocin P7 can be used safely in this concentration range. The antimicrobial activity of bacteriocin P7 was diminished in the presence of CaCl₂ and MgCl₂; however, it was more stable in the presence of other inorganic salts and organic solvents. Furthermore, SDS was shown to enhance the antimicrobial activity of bacteriocin P7. This probably due to the SDS is an anionic surfactant, low concentration of SDS affects the permeability of cell membrane and promotes the signal transmission between cells and cells. Meanwhile, facilitates the secretion of extracellular proteins and polysaccharides and other substances, which are involved in the process of biofilm formation. It is worth mentioning that when studying the effect of organic solvents on bacteriocins, relevant studies also found that SDS increased the activity of bacteriocins (Xi et al., 2018).

These favourable characteristics indicate that this antimicrobial protein may have potential applications in feed processing, drug therapy and other related fields. Bacteriocins can be used in food preservation to treat infections and antibiotic-resistant pathogenic bacteria (Chikindas et al., 2018; Kaškonienė et al., 2017).

With the further development of biotechnology and in-depth research on the structure and function of various proteins, protein isolation and purification techniques have developed rapidly, and chromatography, electrophoresis, molecular blotting and other techniques can be used to isolate target proteins (Liu et al., 2020). For example, the antimicrobial protein PAG14, which was isolated from the metabolites of *B*. *velezensis* G14 via dextran gel and ion chromatography, was classified by LC‒MS/MS as a class III bacteriocin, similar to lysozyme C (Peng et al., 2024). A novel *bacteriocin, LFX01, of Lactobacillus plantarum* LF-8*,* which was isolated from the intestine of tilapia, showed excellent stability under heat and acid‒base stresses and exhibited sensitivity to a variety of enzymes, such as proteinase K, pepsin and trypsin (Jiang et al., 2022). In this study, the bacteriocin was purified via gel chromatography, anion chromatography and RP-HPLC and identified as an amyloliquecidin GF610 beta analogue via LC‒MS/MS (Gerst et al., 2022). Amyloliquecidin GF610 is a two-component wool‒sulfur antimicrobial peptide, and its α and β peptides have single equivalent volume masses of 3026 and 2451 Da, with molecular formulas of C130H191N35O39S5 and C110H158N26O30S4, respectively. Bacteriocin P7 has significant inhibitory effects on a variety of pathogenic bacteria and is a potential antimicrobial drug with broad application prospects.

## 5 Conclusion

The optimal medium composition for bacteriocin production in *B. velezensis* G7 includes 4.5 g/100 mL glucose, 1.5 g/100 mL yeast extract, and 1.2 g/100 mL MgSO_4_·7H_2_O. The CFS exhibited enhanced stability at 40–60°C, 4, −25, and −80°C storage. And remains active under prolonged UV exposure. The bacteriostatic activity remained consistent at pH 6. Among the various substances tested, Proteinase E had the greatest effect on the activity of bacteriocin P7. Furthermore, the class I bacteriocin produced by *B. velezensis* G7 was purified and identified as responsible for the production of bacteriocin P7, which demonstrated a pronounced inhibitory effect on *S. aureus*, with a molecular weight of approximately 6.5–14.4 kDa. Given its stability, broad-spectrum antimicrobial activity, and safety profile, bacteriocin P7 holds potential for applications in food preservation, as a feed additive, or even as a novel antimicrobial agent for controlling infections. Further studies should focus on genetically engineering bacteriocin P7 for large-scale production, as well as evaluating its efficacy in in vivo models to fully assess its potential for industrial and clinical applications.

## Data Availability

The original contributions presented in the study are included in the article/supplementary material, further inquiries can be directed to the corresponding author.

## References

[B1] Acharya S. Jali P. Pradhan M. Pradhan C. Mohapatra P. K. (2023). Antimicrobial and Antioxidant property of a True mangrove Rhizophora apiculata Bl. Chem. & Biodivers. 20 (9), e202201144. 10.1002/cbdv.202201144 37471640

[B2] Ahsan T. Liang C. Yu S. Pei X. Xie J. Lin Y. (2023). Screening and optimization of fermentation medium for *Bacillus velezensis* BP-1 and its biocontrol effects against *Peyronellaea arachidicola* . Appl. Sci. 13 (0), 4653. 10.3390/app13084653

[B3] Banerjee G. Nandi A. Ray A. K. (2017). Assessment of hemolytic activity, enzyme production and bacteriocin characterization of *Bacillus* subtilis LR1 isolated from the gastrointestinal tract of fish. Archives Microbiol. 199 (1), 115–124. 10.1007/s00203-016-1283-8 27590016

[B4] Bastos E. M. Simone M. Jorge D. M. Soares A. E. E. Spivak M. (2008). *In vitro* study of the antimicrobial activity of Brazilian propolis against Paenibacillus larvae. J. Invertebr. pathology 97 (3), 273–281. 10.1016/j.jip.2007.10.007 18054037

[B5] Begyn K. Kim D. T. Heyndrickx M. Michiels C. Aertsen A. Rajkovic A. (2020). Directed evolution by UV-C treatment of *Bacillus* cereus spores. Int. J. Food Microbiol. 317, 108424. 10.1016/j.ijfoodmicro.2019.108424 31790956

[B6] Chen S. Shi F. Liu F. Yang N. Xu X. Jin Y. (2025). Kinetics study on microbial growth and surfactin production of *Bacillus subtilis* ATCC 21332 under the synergistic effect of magnetic field and Mg^2+^ . Food Biosci. 63, 105546. 10.1016/j.fbio.2024.105546

[B7] Chikindas M. L. Weeks R. Drider D. Chistyakov V. A. Dicks L. M. (2018). Functions and emerging applications of bacteriocins. Curr. Opin. Biotechnol. 49, 23–28. 10.1016/j.copbio.2017.07.011 28787641 PMC5799035

[B8] De Leersnyder I. De Gelder L. Van Driessche I. Vermeir P. (2018). Influence of growth media components on the antibacterial effect of silver ions on *Bacillus subtilis* in a liquid growth medium. Sci. Rep. 8 (1), 9325. 10.1038/s41598-018-27540-9 29921908 PMC6008294

[B9] Elazzazy A. M. Mobarki M. O. Baghdadi A. M. Bataweel N. M. Al-Hejin A. M. (2024). Optimization of culture conditions and batch process control for the augmented production of bacteriocin by *Bacillus* species. Microorganisms 12 (4), 651. 10.3390/microorganisms12040651 38674596 PMC11051734

[B10] Fernandes A. Jobby R. (2022). Bacteriocins from lactic acid bacteria and their potential clinical applications. Appl. Biochem. Biotechnol. 194 (10), 4377–4399. 10.1007/s12010-022-03870-3 35290605

[B11] Friess D. A. (2016). Mangrove forests. Curr. Biol. 26 (16), R746–R748. 10.1016/j.cub.2016.04.004 27554647

[B12] Gerst M. M. Somogyi Á. Yang X. Yousef A. E. (2022). Detection and characterization of a rare two-component lantibiotic, Amyloliquecidin GF610 produced by *Bacillus velezensis*, using a combination of culture, molecular and bioinformatic analyses. J. Appl. Microbiol. 132 (2), 994–1007. 10.1111/jam.15290 34487591

[B13] Gomes R. J. Ida E. I. Spinosa W. A. (2022). Nutritional supplementation with amino acids on bacterial cellulose production by Komagataeibacter intermedius: effect analysis and application of response surface methodology. Appl. Biochem. Biotechnol. 194 (11), 5017–5036. 10.1007/s12010-022-04013-4 35687307

[B14] Guo Z. Gai C. Cai C. Chen L. Liu S. Zeng Y. (2017). Metabolites with insecticidal activity from Aspergillus fumigatus JRJ111048 isolated from mangrove plant *Acrostichum specioum* endemic to Hainan Island. Mar. Drugs 15 (12), 381. 10.3390/md15120381 29211003 PMC5742841

[B15] Hwanhlem N. Biscola V. El-Ghaish S. Jaffrès E. Dousset X. Haertlé T. (2013). Bacteriocin-producing lactic acid bacteria isolated from mangrove forests in Southern Thailand as potential bio-control agents: Purification and characterization of bacteriocin produced by Lactococcus lactis subsp. lactis KT2W2L. Probiotics Antimicrob. proteins 5 (4), 264–278. 10.1007/s12602-013-9150-2 26783072

[B16] Idris A. L. Li W. Huang F. Lin F. Guan X. Huang T. (2024). Impacts of UV radiation on *Bacillus* biocontrol agents and their resistance mechanisms. World J. Microbiol. & Biotechnol. 40 (2), 58. Published 2024 Jan 2. 10.1007/s11274-023-03856-1 38165488

[B17] Islam T. Rabbee M. F. Choi J. Baek K. H. (2022). Biosynthesis, molecular regulation, and application of Bacilysin produced by *Bacillus* species. Metabolites 12 (5), 397. 10.3390/metabo12050397 35629901 PMC9147277

[B18] Jalali E. Maghsoudi S. Noroozian E. (2020). A novel method for biosynthesis of different polymorphs of TiO2 nanoparticles as a protector for *Bacillus* thuringiensis from Ultra Violet. Sci. Rep. 10 (1), 426. 10.1038/s41598-019-57407-6 31949264 PMC6965098

[B19] Jiang Y. H. Xin W. G. Zhang Q. L. Lin L. B. Deng X. Y. (2022). A novel bacteriocin against *Shigella flexneri* from *Lactiplantibacillus plantarum* isolated from Tilapia intestine: purification, antibacterial properties and antibiofilm activity. Front. Microbiol. 12, 779315. 10.3389/fmicb.2021.779315 35069481 PMC8769287

[B20] Kamoun F. Zouari N. Saadaoui I. Jaoua S. (2009). Improvement of *Bacillus* thuringiensis bacteriocin production through culture conditions optimization. Prep. Biochem. & Biotechnol. 39 (4), 400–412. 10.1080/10826060903209653 19739026

[B21] Kaškonienė V. Stankevičius M. Bimbiraitė-Survilienė K. Naujokaitytė G. Šernienė L. Mulkytė K. (2017). Current state of purification, isolation and analysis of bacteriocins produced by lactic acid bacteria. Appl. Microbiol. Biotechnol. 101 (4), 1323–1335. 10.1007/s00253-017-8088-9 28070665

[B22] Kaur R. Tiwari S. K. (2018). Membrane-acting bacteriocin purified from a soil isolate *Pediococcus pentosaceus* LB44 shows broad host-range. Biochem. biophysical Res. Commun. 498 (4), 810–816. 10.1016/j.bbrc.2018.03.062 29530530

[B23] Lahiri D. Nag M. Dutta B. Sarkar T. Pati S. Basu D. (2022). Bacteriocin: A natural approach for food safety and food security. Front. Bioeng. Biotechnol. 10, 1005918. 10.3389/fbioe.2022.1005918 36353741 PMC9637989

[B24] Lajis A. F. B. (2020). Biomanufacturing process for the production of bacteriocins from Bacillaceae family. Bioresour. Bioprocess. 7 (1), 8–4365. 10.1186/s40643-020-0295-z

[B25] León Madrazo A. Fuentes Ortíz A. B. Morales Mendoza L. F. Segura Campos M. R. (2022). Antibacterial peptide fractions from chia seeds (Salvia hispanica L.) and their stability to food processing conditions. J. food Sci. Technol. 59 (11), 4332–4340. 10.1007/s13197-022-05506-0 36193479 PMC9525467

[B26] Li Y. Wang Y. Liu Y. Li X. Feng L. Li K. (2022). Optimization of an economical medium composition for the coculture of *Clostridium butyricum* and *Bacillus coagulans* . Amb. Express 12 (1), 19. 10.1186/s13568-022-01354-5 35166947 PMC8847521

[B27] Li Y. Wu Y. Peng Z. Long L. Guo Q. Tian L. (2024). Isolation and identification of bacteriocin-producing lactic acid bacteria from Daqu and mining of bacteriocin gene. Biologa 79, 2891–2905. 10.1007/s11756-024-01746-x

[B28] Liu S. Li Z. Yu B. Wang S. Shen Y. Cong H. (2020). Recent advances on protein separation and purification methods. Adv. colloid interface Sci. 284, 102254. 10.1016/j.cis.2020.102254 32942182

[B29] Ma H. Ding Y. Peng J. Li Y. Pan R. Long Y. (2025). Identification and characterization of a novel bacteriocin PCM7-4 and its antimicrobial activity against *Listeria monocytogenes* . Microbiol. Res. 290, 127980. 10.1016/j.micres.2024.127980 39581173

[B30] Ming S. Chen X. Zhang N. Li S. Zhu Z. Cheng S. (2022). Structure and stability analysis of antibacterial substance produced by selenium enriched *Bacillus cereus* BC1. Archives Microbiol. 204 (3), 196. 10.1007/s00203-022-02798-w 35217921

[B31] Owusu-Darko R. Allam M. Ismail A. Ferreira C. A. S. Oliveira S. D. d. Buys E. M. (2020). Comparative genome analysis of *Bacillus sporothermodurans* with its closest phylogenetic neighbor, *Bacillus oleronius*, and *Bacillus cereus* and *Bacillus subtilis* groups. Microorganisms 8 (8), 1185. 10.3390/microorganisms8081185 32759699 PMC7464528

[B32] Peng J. Xie X. Fan T. Ma H. Li Y. Luo S. (2024). Optimization of culture conditions for endophytic bacteria in mangrove plants and isolation and identification of bacteriocin. Front. Pharmacol. 15, 1429423. 10.3389/fphar.2024.1429423 39156104 PMC11327053

[B33] Selvam D. Thangarasu A. Shyu D.J. H. Neelamegam R. Muthukalingan K. Nagarajan K. (2021). Antimicrobial substance produced by *Pseudomonas aeruginosa* isolated from slaughterhouse sediment: physicochemical characterization, purification, and identification. Int. J. Peptide Res. Ther. 27, 887–897. 10.1007/s10989-020-10135-2

[B34] Singh V. Haque S. Niwas R. Srivastava A. Pasupuleti M. Tripathi C. K. M. (2017). Strategies for fermentation medium optimization: An in-depth review. Front. Microbiol. 7, 2087. Published 2017 Jan 6. 10.3389/fmicb.2016.02087 28111566 PMC5216682

[B35] Six A. Mosbahi K. Barge M. Kleanthous C. Evans T. Walker D. (2021). Pyocin efficacy in a murine model of *Pseudomonas aeruginosa* sepsis. J. Antimicrob. Chemother. 76 (9), 2317–2324. 10.1093/jac/dkab199 34142136 PMC8361349

[B36] Sulaiman M. Nissapatorn V. Rahmatullah M. Paul A. K. Rajagopal M. Rusdi N. A. (2022). Antimicrobial secondary metabolites from the mangrove plants of Asia and the Pacific. Mar. drugs 20 (10), 643. 10.3390/md20100643 36286466 PMC9605323

[B37] Vaca J. Ortiz A. Sansinenea E. (2022). A study of bacteriocin like substances comparison produced by different species of *Bacillus* related to *B. cereus* group with specific antibacterial activity against foodborne pathogens. Archives Microbiol. 205 (1), 13. 10.1007/s00203-022-03356-0 36463345

[B38] Vlajkov V. Anđelić S. Pajčin I. Grahovac M. Budakov D. Jokić A. (2022b). Medium for the production of *Bacillus*-based biocontrol agent effective against aflatoxigenic *Aspergillus flavus*: Dual approach for modelling and optimization. Microorganisms 10 (6), 1165. 10.3390/microorganisms10061165 35744682 PMC9228200

[B39] Vlajkov V. Pajčin I. Loc M. Budakov D. Dodić J. Grahovac M. (2022a). The effect of cultivation conditions on Antifungal and maize seed germination activity of *Bacillus*-based biocontrol agent. Bioeng. Basel, Switz. 9 (12), 797. 10.3390/bioengineering9120797 PMC9774550 36551004

[B40] Wang H. Li X. Wang Y. Tao Y. Lu S. Zhu X. (2018). Improvement of n-caproic acid production with *Ruminococcaceae* bacterium CPB6: selection of electron acceptors and carbon sources and optimization of the culture medium. Microb. cell factories 17 (1), 99. 10.1186/s12934-018-0946-3 PMC6019802 29940966

[B41] Wang H. Wang L. Zhang F. Li X. Wang S. Gao D. (2024b). ParalichenysinDY4, a novel bacteriocin-like substance, is employed to control *Clostridium perfringens* . Int. J. Biol. Macromol. 279 (Pt 4), 135412. 10.1016/j.ijbiomac.2024.135412 39245094

[B42] Wang T. Zheng J. Dong S. Ismael M. Shan Y. Wang X. (2022). *Lacticaseibacillus rhamnosus* LS8 Ameliorates Azoxymethane/dextran sulfate sodium-induced colitis-associated tumorigenesis in mice via regulating gut microbiota and inhibiting inflammation. Probiotics Antimicrob. proteins 14 (5), 947–959. 10.1007/s12602-022-09967-9 35788907

[B43] Wang Z. Zhang W. Wang Z. Zhang Z. Liu Y. Liu S. (2024a). Analysis of antimicrobial biological activity of a marine *Bacillus velezensis* NDB. Archives Microbiol. 206 (3), 131. 10.1007/s00203-024-03861-4 38421449

[B44] Wayah S. B. Philip K. (2018). Characterization, yield optimization, scale up and biopreservative potential of fermencin SA715, a novel bacteriocin from *Lactobacillus fermentum* GA715 of goat milk origin. Microb. cell factories 17 (1), 125. 10.1186/s12934-018-0972-1 PMC6090665 30103750

[B45] Xi Q. Wang J. Du R. Zhao F. Han Y. Zhou Z. (2018). Purification and characterization of bacteriocin produced by a strain of *Enterococcus faecalis* TG2. Appl. Biochem. Biotechnol. 184 (4), 1106–1119. 10.1007/s12010-017-2614-1 28952066

[B46] Zhao C. Yang Q. Zhang H. (2017). Optimization of microbial flocculant-producing medium for *Bacillus* subtilis. Indian J. Microbiol. 57 (1), 83–91. 10.1007/s12088-016-0631-3 28148983 PMC5243253

[B47] Zhao J. Zhou Z. Bai X. Zhang D. Zhang L. Wang J. (2022). A novel of new class II bacteriocin from *Bacillus velezensis* HN-Q-8 and its antibacterial activity on *Streptomyces scabies* . Front. Microbiol. 13, 943232. Published 2022 Jul 29. 10.3389/fmicb.2022.943232 35966655 PMC9372549

